# A corona-like distribution and patchy pattern of cerebellar infarcts identify patients with giant cell arteritis

**DOI:** 10.1177/17562864251405203

**Published:** 2026-02-04

**Authors:** Carolin Beuker, Jan-Kolja Strecker, Veith Jungmann, Nils Werring, Tobias Brix, Christian Thomas, Maximilian Christian Wankner, Antje Schmidt-Pogoda, Paul Stracke, Bernd Eckert, Thomas Raphael Meinel, Marcel Arnold, Jens Schaumberg, Schulamith Krüger, Milani Deb-Chatterji, Christina Krüger, Tim Magnus, Joachim Röther, Jens Minnerup

**Affiliations:** Department of Neurology, University of Münster, Albert-Schweitzer-Campus 1, Gebäude A1, Münster 48149, Germany; Department of Neurology, University Hospital Schleswig-Holstein and University of Lübeck, Lübeck, Germany; Department of Neurology, University of Münster, Münster, Germany; Department of Neurology, University Hospital Schleswig-Holstein and University of Lübeck, Lübeck, Germany; Institute of Medical Informatics, University of Münster, Münster, Germany; Institute of Neuropathology, University Hospital Münster, Münster, Germany; Department of Neurosurgery, University Hospital Cologne, Cologne, Germany; Department of Neurology, University of Münster, Münster, Germany; Department of Clinical Radiology, University Hospital of Münster, Münster, Germany; Department of Neuroradiology, Asklepios Klinik Hamburg Altona, Hamburg, Germany; Department of Neurology, Inselspital, Bern University Hospital and University of Bern, Bern, Switzerland; Department of Neurology, Inselspital, Bern University Hospital and University of Bern, Bern, Switzerland; Department of Neurology, Community Hospital Sana Kliniken Lübeck, Lübeck, Germany; Department of Neurology, Helios Klinikum Uelzen, Uelzen, Germany; University Hospital Schleswig-Holstein Campus Kiel, Kiel, Germany; Department of Neurology, University Hospital Hamburg-Eppendorf, Hamburg, Germany; Department of Neurology, University Hospital Hamburg-Eppendorf, Hamburg, Germany; Department of Neurology, Asklepios Klinik Hamburg Altona, Hamburg, Germany; Department of Neurology, University of Münster, Münster, Germany; Department of Neurology, University Hospital Schleswig-Holstein and University of Lübeck, Lübeck, Germany

**Keywords:** cerebellar, GCA, infarction, intracranial, pattern

## Abstract

**Background:**

Cerebrovascular events are a potentially serious complication of giant cell arteritis (GCA) with intracranial involvement. However, diagnosing GCA in this context remains challenging, as classical clinical features may be absent.

**Objectives:**

To identify characteristic cerebellar infarct patterns associated with intracranial GCA and to differentiate them from other common causes of posterior circulation stroke.

**Design:**

Multicenter retrospective study.

**Methods:**

A total of 125 patients with cerebellar infarctions of various etiologies were included. Among these, 19 patients had confirmed intracranial GCA. Infarct patterns were compared to those seen in strokes of cardioembolic origin (*n* = 42), arterio-arterial embolism from proximal vertebral artery atherosclerosis (*n* = 13), local atherosclerotic stenosis of the V4 segment (*n* = 21), and vertebral artery dissection (*n* = 30). Infarct topography was assessed using acute-phase diffusion-weighted magnetic resonance imaging. Sensitivity and specificity were calculated for individual imaging features.

**Results:**

Distinct imaging signatures were observed in patients with GCA. A “corona-like” infarct pattern, defined by sparing of the medial branch of the proximal posterior inferior cerebellar artery (PICA), demonstrated a sensitivity of 79% and a specificity of 64%. A patchy infarct pattern, characterized by scattered non-confluent lesions, was present in 53% of GCA cases and showed high specificity (93%). When both features were present, specificity increased to 98% and sensitivity was reduced to 47%.

**Conclusion:**

Our findings reveal a distinct cerebellar infarct pattern associated with intracranial GCA, characterized by a corona-like configuration and patchy lesions predominantly involving the lateral PICA territory. Recognition of this imaging phenotype may enhance diagnostic accuracy in challenging cases and facilitate the timely initiation of immunosuppressive therapy.

## Introduction

Giant cell arteritis (GCA) is the most prevalent idiopathic systemic vasculitis affecting large- and medium-sized arteries.^1,2^ The underlying vessel wall inflammation can result in luminal narrowing, occlusion, or aneurysm formation.^3^ In approximately 4% of cases, these vascular changes extend to intracranial arteries, potentially causing severe and recurrent ischemic strokes.^4–6^ Diagnosing GCA becomes particularly challenging when intracranial involvement is the initial clinical manifestation. In such cases, hallmark systemic symptoms, such as headache and elevated erythrocyte sedimentation rate, are often absent.^6^ Consequently, stroke may be the presenting feature, and an underlying vasculitic etiology may not be immediately considered, especially given the advanced age of most affected patients and the prevalence of alternative stroke mechanisms in this population.^6^ Differentiating vasculitis-related arterial pathology from other stroke etiologies remains difficult, particularly in the posterior circulation.

Recent studies have proposed a distinctive vascular imaging pattern suggestive of intracranial GCA, characterized by segmental stenoses of the vertebral arteries with preferential involvement of the distal segment prior to the posterior inferior cerebellar artery (PICA) origin, together with long-segment arterial involvement consistent with the so-called “slope sign” known from axillary artery affection in GCA, which may aid in select diagnostic scenarios.^6,7^ While these insights have enhanced the understanding of intracranial GCA, substantial diagnostic uncertainty persists. This underscores the need to refine imaging-based criteria that can support earlier and more accurate identification of the disease. Against this background, the present study aimed to identify a characteristic cerebellar infarct pattern associated with GCA and to evaluate its diagnostic utility in comparison with other common causes of cerebellar stroke.

## Methods

### Study population and design

This multicenter retrospective study included patients with cerebellar infarctions due to intracranial vasculitic involvement of GCA. Participants were recruited from the Departments of Neurology at University Hospital Münster, Asklepios Clinic Hamburg-Altona, HELIOS Hospital Uelzen, University Medical Center Hamburg-Eppendorf, and University Hospital Bern.

Patients with intracranial GCA were identified retrospectively across all participating centers between January 2008 and May 2022. The diagnosis of GCA with intracranial involvement was made by the treating neurologists in accordance with the American College of Rheumatology classification criteria.^8^ Confirmation was obtained either through temporal artery biopsy (*n* = 7) or through imaging evidence consistent with vasculitis, specifically, ultrasound of the superficial temporal artery (*n* = 12). Additional inclusion criteria comprised: a cerebellar infarction with a clearly established etiology, and availability of acute-phase diffusion-weighted MRI (DWI).

To identify clinical and radiological features specific to intracranial GCA, 19 patients with GCA-associated cerebellar infarcts were compared to a control cohort of 106 patients with cerebellar infarctions of other etiologies. The control group comprised all consecutive non-GCA cerebellar infarctions admitted to the Department of Neurology, University Hospital Münster, between January 2015 and May 2022.

Patients were excluded if they had non-acute cerebellar infarctions, lacked DWI, or had an unclear stroke etiology. A detailed overview of the patient selection process is provided in Supplemental Figure 1.

### Standard protocol approvals, registrations, and patient consents

The study was conducted in accordance with the Declaration of Helsinki and approved by the Ethics Committee Westfalen-Lippe (No. 2022-515-f-S). Written informed consent was not necessary because the study was performed retrospectively by screening patient files.

### MRI data acquisition and analysis

All patients underwent diffusion-weighted magnetic resonance imaging (DWI-MRI) during the acute phase of cerebellar infarction. Advanced vessel wall imaging using black-blood MRI was not performed systematically, as this technique was not routinely available and lacked standardization across participating centers. Infarct regions were identified based on hyperintense signals indicative of restricted diffusion. Lesions were independently evaluated by two investigators (C.B. and J.M.), who were blinded to the underlying etiology during the assessment. Any discrepancies were resolved by consensus. A “patchy” infarct pattern was defined as the presence of two or more spatially distinct, non-confluent lesions within the same vascular territory. Interrater agreement for the presence of a patchy pattern was assessed using Cohen’s kappa coefficient (κ = 0.78), indicating substantial agreement. To ensure standardized topographic analysis, the cerebellum was divided into four axial levels (from rostral to caudal), allowing for a comprehensive and anatomically consistent evaluation of infarct distribution across all patients and etiologic subgroups. For each patient, individual lesion maps were created and subsequently aggregated by stroke etiology to generate group-level infarct heatmaps. These visualizations captured the frequency and distribution of cerebellar infarcts within each subgroup. All image processing and overlay visualizations were performed using Adobe Illustrator CS5 (Adobe Inc., San Jose, CA, USA).

### Statistical analysis

Categorical variables are presented as absolute counts and percentages. Continuous variables were tested for normality using the Shapiro–Wilk test. Normally distributed variables are reported as means with standard deviations (SD), whereas non-normally distributed variables are presented as medians with ranges. Descriptive and comparative analyses were performed using GraphPad Prism version 8 (GraphPad Software, La Jolla, CA, USA). Depending on the distribution, comparisons between groups were performed using either the Student’s *t* test or the Mann–Whitney *U* test. For categorical variables, Fisher’s exact test was applied due to small subgroup sizes. A two-tailed *p* value of <0.05 was considered statistically significant. Sensitivity and specificity for each imaging marker were calculated using 2 × 2 contingency tables comparing GCA-related infarcts with those from other etiologies. Additionally, the proportion of positive findings across all diagnostic subgroups was assessed. Diagnostic accuracy metrics were calculated using R version 4.3.2 (R Foundation for Statistical Computing, Vienna, Austria).

### Data availability statement

Anonymized summary data will be shared by reasonable request from any qualified investigator.

## Results

### Patient baseline characteristics

Baseline demographic and clinical characteristics of the study population are summarized in Table 1. The cohort included 19 patients with GCA-associated cerebellar infarctions attributed to intracranial vasculitis. For comparison, cerebellar infarctions of alternative etiologies were analyzed, comprising cardioembolic strokes (*n* = 42), arterio-arterial embolism from proximal atherosclerotic vertebral artery stenosis (*n* = 13), local thrombotic occlusion due to atherosclerotic V4 segment stenosis (*n* = 21), and vertebral artery dissection (*n* = 30). Patients with GCA had a mean age of 74.3 years (SD 5.4), representing the oldest subgroup. In contrast, those with vertebral artery dissection were significantly younger, with a mean age of 42.4 years. Female sex predominated in the GCA (57.9%) and dissection (53.3%) groups, whereas the arterio-arterial embolism subgroup was predominantly male (only 23.1% female). The distribution of vascular risk factors varied across etiologies. Hypertension was most prevalent among patients with cardioembolic strokes (83.3%) and those with local thrombotic occlusion due to V4 atherosclerosis (85.7%), while markedly less common in patients with dissection (16.7%). At hospital admission, the median NIH Stroke Scale (NIHSS) score in the GCA group was 3 (range 1–5.5). Importantly, neurological impairment as assessed by the NIHSS remained elevated from admission to discharge in the GCA group (median NIHSS 2, range 1–12.5), indicating a more persistent or multifocal neurological burden compared to other etiologies, where most patients exhibited minimal or no progression.

**Table 1.** Baseline characteristics of study population.

Variables	Patients with GCA (*n* = 19)	Patients with cardioembolic origin (*n* = 42)	Patients with arterio-arterial embolism due to proximal atherosclerotic VA stenosis (*n* = 13)	Patients with local atherosclerotic V4 stenosis (*n* = 21)	Patients with vertebral artery dissection (*n* = 30)	*p* Value*
Demographics						
Age, mean (SD), years	74.3 (5.4)	70 (16.1)	65.4 (13.5)	70.4 (11.0)	42.4 (11.1)	<0.001
Women, *n* (%)	11 (57.9)	19 (45.2)	3 (23.1)	7 (33.3)	16 (53.3)	0.22
Comorbidities, *n* (%)						
Hypertension	12/17 (63.2)	35/42 (83.3)	9/13 (69.2)	18/21 (85.7)	5/30 (16.7)	0.79
Diabetes mellitus	4/17 (21.1)	7/42 (16.7)	2/13 (15.4)	7/21 (33.3)	3/30 (10)	0.52
Nicotine abuse	3/17 (15.8)	7/42 (16.7)	3/13 (23.1)	4/21 (19.0)	2/30 (6.7)	0.73
Hyperlipidemia	4/17 (21.1)	19/42 (45.2)	3/13 (23.1)	7/21 (33.3)	8/30 (26.7)	0.42
Atrial fibrillation	2/17 (10.5)	36/42 (85.7)	0/0 (0)	0/0 (0)	0/0 (0)	0.03
NIHSS score, median (range)						
At onset	3 (1–5.5)	3 (0–5)	3 (1–5)	0 (0–2)	2 (0–6)	<0.001
At disease course	2 (1–12.5)	0 (0–2)	2 (1–14)	0 (0–1)	0 (0–2)	<0.001

* *p* Values for comparisons between GCA and non-GCA groups.

GCA, giant cell arteritis; NIHSS, NIH Stroke Scale; SD, standard deviation.

### Topographic distribution of cerebellar infarcts

**Figure 1.**
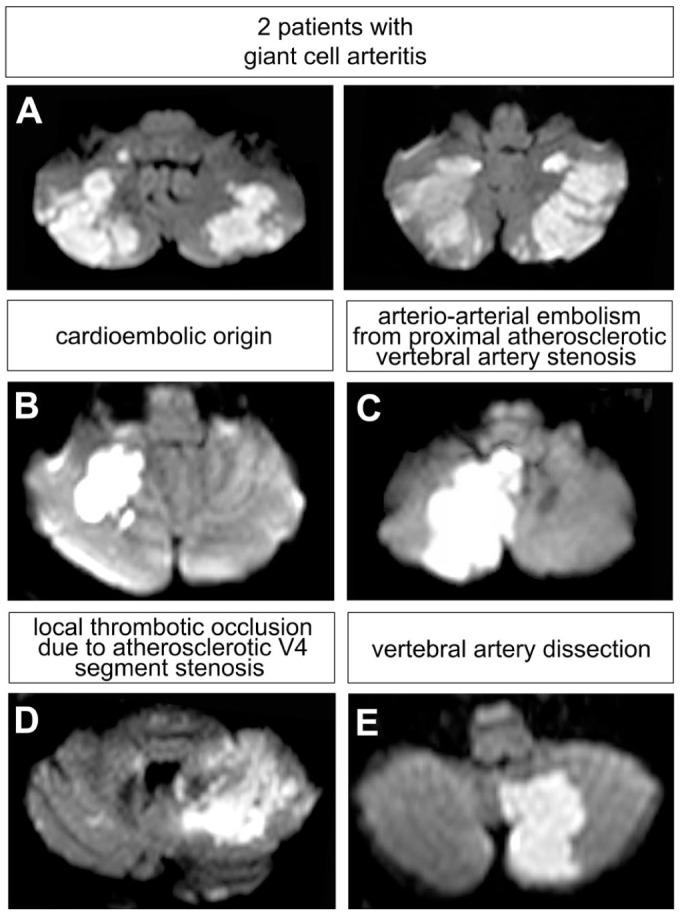
Axial diffusion-weighted MRI (DWI) scans of cerebellar infarctions of different etiologies. (a) Infarct distribution in patients with GCA and intracranial involvement (*n* = 2). (b) Representative infarct distribution in a patient with cardioembolic origin. (c) Representative infarct distribution in a patient with arterio-arterial embolism due to proximal atherosclerotic vertebral artery stenosis. (d) Representative infarct distribution in a patient with local atherosclerotic V4 stenosis. (e) Representative infarct distribution in a patient with vertebral artery dissection. GCA, giant cell arteritis.

To highlight the characteristic imaging features of GCA-related strokes, Figure 1 displays axial DWI-MRI slices from two representative patients with intracranial GCA, alongside examples from other stroke etiologies. In GCA patients, infarcts typically presented as small, patchy, non-confluent lesions predominantly affecting the lateral territory of the PICA, while sparing regions supplied by the medial PICA (Figure 1(a); Supplemental Table 1). These lesions exhibited a peripheral distribution aligned with cerebellar folia and were bilaterally distributed in more than half of the cases (58%). In contrast, infarcts observed in patients with non-GCA etiologies were generally more confluent and involved both proximal and distal cerebellar territories (Figure 1(b)–(e)).

**Figure 2.**
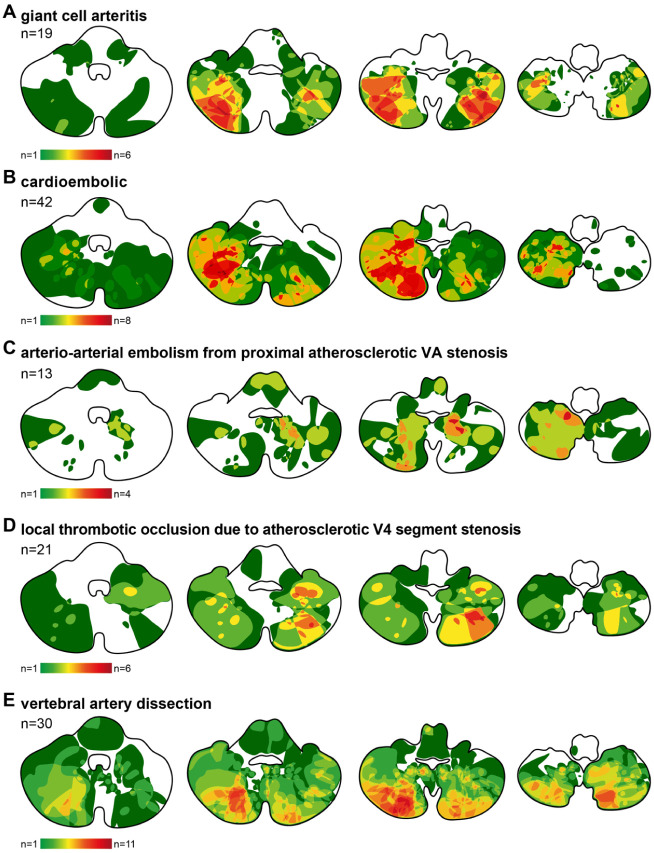
Heatmaps illustrating the distribution of cerebellar infarcts across four standardized axial levels of the cerebellum. (a) Infarct distribution in patients with GCA and intracranial involvement (*n* = 19). (b) Infarct distribution in patients with cardioembolic origin (*n* = 41). (c) Infarct distribution in patients with arterio-arterial embolism due to proximal atherosclerotic vertebral artery stenosis (*n* = 13). (d) Infarct distribution in patients with local atherosclerotic V4 stenosis (*n* = 21). (e) Infarct distribution in patients with vertebral artery dissection (*n* = 30). Color intensity reflects frequency of infarction at a given location within the respective group. GCA, giant cell arteritis.

Figure 2 provides an overview of infarct topographies across all etiologies using aggregated DWI-based heatmaps. Patients with GCA consistently showed infarcts restricted to the lateral PICA territory, reflecting preferential involvement of terminal vascular zones typical of vasculitic pathology (Figure 2(a)). Importantly, the medial PICA region was characteristically spared, resulting in a corona-like distribution pattern. In contrast, patients with strokes from other causes exhibited infarct patterns that frequently encompassed both lateral and medial PICA regions (Figure 2(b)–(e)).

### Diagnostic accuracy of GCA-related strokes

To evaluate the diagnostic utility of specific MRI features, we assessed the following markers: (1) sparing of the medial PICA territory—corresponding to a corona-like pattern—and (2) a patchy infarction pattern. We also analyzed the diagnostic performance of their combined presence. Sensitivity and specificity were determined for each feature in differentiating GCA from other stroke etiologies (Table 2). In addition, positive and negative predictive values (PPV and NPV) were calculated to further illustrate the diagnostic accuracy of these imaging markers. Sparing of the medial PICA territory demonstrated the highest sensitivity (79%) with a moderate specificity (64%) and a high NPV (94%). In contrast, the patchy infarction pattern showed lower sensitivity (53%) but markedly higher specificity (93%) and an NPV of 92%, indicating strong diagnostic value when present. The combination of both features yielded the highest specificity (98%) and PPV (82%) but lower sensitivity (47%). This suggests that while the concurrent presence of both findings is highly confirmatory for GCA, their absence does not reliably exclude the diagnosis. To control for potential confounding by demographic and vascular factors, we performed a multivariable logistic regression analysis including age, sex, and vascular risk factors (Supplemental Table 2). The combined presence of both infarct patterns remained independently associated with GCA-related stroke (*p* = 0.003).

**Table 2.** Diagnostic accuracy of MRI features for differentiating GCA-related stroke from other etiologies.

Feature	Sensitivity (%)	95% CI	Specificity (%)	95% CI	PPV (%)	95% CI	NPV (%)	95% CI
Corona-like pattern	79	[0.567, 0.915]	64	[0.547, 0.726]	28	[0.180, 0.416]	94	[0.866, 0.978]
Patchy pattern	53	[0.317, 0.727]	93	[0.870, 0.968]	59	[0.360, 0.784]	92	[0.849, 0.956]
Both features present	47	[0.273, 0.683]	98	[0.934, 0.995]	82	[0.523, 0.949]	91	[0.846, 0.952]

GCA, giant cell arteritis; NPV, negative predictive value; PPV, positive predictive value.

## Discussion

In this multicenter retrospective study, we systematically analyzed infarct patterns in patients with GCA-associated cerebellar stroke and compared them with four common alternative stroke etiologies. Our findings reveal a distinct topographic signature indicative of intracranial GCA, with important clinical implications. We identified the following key imaging characteristics: (1) A corona-like pattern, defined by sparing of the medial PICA territory, emerged as the most sensitive marker for GCA-related infarction, present in nearly 80% of cases, (2) a patchy infarction pattern demonstrated high specificity for GCA, and (3) the co-occurrence of both imaging features yielded the highest overall specificity, supporting their diagnostic utility in distinguishing GCA from other stroke mechanisms.

Consistent with previous studies, our data confirm that involvement of the posterior circulation in intracranial GCA represents a clinically serious and impactful manifestation.^9,10^ Furthermore, our findings reinforce earlier observations that this involvement is frequently bilateral.^10^ However, our study extends the current knowledge by offering the first systematic, etiology-specific comparison of cerebellar infarct patterns in GCA. While an increasing number of reports, including systematic reviews, address intracranial manifestations of GCA,^9,11,12^ infarct pattern characterization has not been a focus of prior investigations. From a pathophysiological standpoint, our data support the hypothesis that the sparing of medial arterial segments reflects the segmental nature of large-vessel vasculitis. In contrast, infarcts due to atherosclerotic disease more commonly involve both medial and lateral PICA territories, reflecting the predilection of atherosclerosis for branch points and proximal vessel segments exposed to turbulent flow and shear stress. As a result, atherosclerotic lesions typically occur at the origin of the PICA, where both the medial and lateral branches arise almost simultaneously, often leading to larger, confluent infarcts encompassing multiple subterritories. In GCA, by contrast, the segmental nature of vasculitic involvement results in a more selective pattern. The preferential involvement of lateral PICA-supplied regions in our cohort could further reflect anatomical or hemodynamic factors, such as a more peripheral vascular supply and limited collateralization, although this remains speculative.

The imaging pattern identified in this study may serve as a useful diagnostic clue in cases where clinical features alone are insufficient to establish the diagnosis. Early recognition of this pattern is crucial for initiating prompt immunosuppressive therapy. The observed MRI findings may guide further diagnostic evaluation, including vascular imaging (Magnetic Resonance Angiography, MRA or Computed Tomography Angiohgraphy, CTA) to detect the characteristic intracranial vascular pattern as previously described by our group,^6^ as well as temporal artery ultrasound and, if indicated, temporal artery biopsy.

Although partially patchy infarct configurations may also be observed in other stroke etiologies, including arterio-arterial embolism or cardioembolic infarctions, these typically appear larger, more confluent, and less sharply demarcated. In our cohort, the patchy pattern was most frequent and most pronounced in GCA, characterized by multiple small, non-confluent lesions with distinct borders and distal PICA predominance, reflecting the segmental nature of vasculitic vessel involvement.

Several vasculitides and vasculopathies can mimic intracranial GCA, including amyloid-β–related angiitis (ABRA/amyloid angiitis), primary angiitis of the CNS (PACNS), and varicella-zoster virus (VZV) vasculopathy. ABRA typically affects older adults and may show leptomeningeal enhancement and microhemorrhages suggestive of cerebral amyloid angiopathy^13^; PACNS often presents at a younger age with headache, cognitive/behavioral change, and multifocal deficits, with cerebrospinal fluid (CSF) pleocytosis in the majority^14^; VZV vasculopathy may be preceded by zoster and can be supported by intrathecal anti-VZV antibodies or polymerase chain reaction (PCR).^15^ Although patchy, multifocal posterior circulation lesions may occur in these entities, our cohort’s predominant lateral PICA involvement with sparing of the medial PICA (“corona-like” pattern), together with the broader clinical context and extracranial large-vessel involvement characteristic of GCA, provides converging diagnostic evidence. Nonetheless, in equivocal cases, additional testing, CSF VZV studies, or biopsy should be considered.

Key strengths of this study include its systematic, etiology-based comparative approach, multicenter design, and standardized imaging analysis. Limitations include the relatively small number of GCA cases, which reflects the rarity of intracranial involvement. This retrospective, multicenter design entails heterogeneity in MRI protocols, which may influence lesion detection and the appearance of “patchy” or “corona-like” patterns. Although two blinded raters with consensus adjudication were used, misclassification bias cannot be excluded. In addition, the retrospective design may introduce inherent selection bias. Moreover, the control cohort originated from a single center, which may further contribute to selection bias and limit the generalizability of the comparative findings. Nevertheless, the inclusion of all consecutive, well-characterized cases enhances the robustness and generalizability of our findings.

## Conclusion

Cerebellar infarcts due to intracranial GCA exhibit a distinctive imaging phenotype, characterized by a corona-like pattern sparing the medial PICA territory and the presence of patchy lesions. This constellation may serve as a valuable diagnostic marker, supporting early recognition and guiding both diagnosis and treatment.

## Supplemental Material

sj-docx-2-tan-10.1177_17562864251405203 – Supplemental material for A corona-like distribution and patchy pattern of cerebellar infarcts identify patients with giant cell arteritis Supplemental material, sj-docx-2-tan-10.1177_17562864251405203 for A corona-like distribution and patchy pattern of cerebellar infarcts identify patients with giant cell arteritis by Carolin Beuker, Jan-Kolja Strecker, Veith Jungmann, Nils Werring, Tobias Brix, Christian Thomas, Maximilian Christian Wankner, Antje Schmidt-Pogoda, Paul Stracke, Bernd Eckert, Thomas Raphael Meinel, Marcel Arnold, Jens Schaumberg, Schulamith Krüger, Milani Deb-Chatterji, Christina Krüger, Tim Magnus, Joachim Röther and Jens Minnerup in Therapeutic Advances in Neurological Disorders

sj-pdf-1-tan-10.1177_17562864251405203 – Supplemental material for A corona-like distribution and patchy pattern of cerebellar infarcts identify patients with giant cell arteritis Supplemental material, sj-pdf-1-tan-10.1177_17562864251405203 for A corona-like distribution and patchy pattern of cerebellar infarcts identify patients with giant cell arteritis by Carolin Beuker, Jan-Kolja Strecker, Veith Jungmann, Nils Werring, Tobias Brix, Christian Thomas, Maximilian Christian Wankner, Antje Schmidt-Pogoda, Paul Stracke, Bernd Eckert, Thomas Raphael Meinel, Marcel Arnold, Jens Schaumberg, Schulamith Krüger, Milani Deb-Chatterji, Christina Krüger, Tim Magnus, Joachim Röther and Jens Minnerup in Therapeutic Advances in Neurological Disorders

## Declarations

**Ethics approval and consent to participate:** The study was conducted in accordance with the Declaration of Helsinki and approved by the Ethics Committee Westfalen-Lippe (No. 2022-515-f-S). Written informed consent was not necessary because the study was performed retrospectively by screening patient files.

**Consent for publication:** Not applicable.

**Availability of data and materials:** Anonymized summary data will be shared by reasonable request from any qualified investigator.
