# Supplementary figures and images for: A corona-like distribution and patchy pattern of cerebellar infarcts identify patients with giant cell arteritis
Source: Ther Adv Neurol Disord. 2026 Feb 4;19:17562864251405203. doi: 10.1177/17562864251405203 (PMC12873070; doi:10.1177/17562864251405203)

**Supplemental Figure 1:** Flow diagram illustrating patient selection.

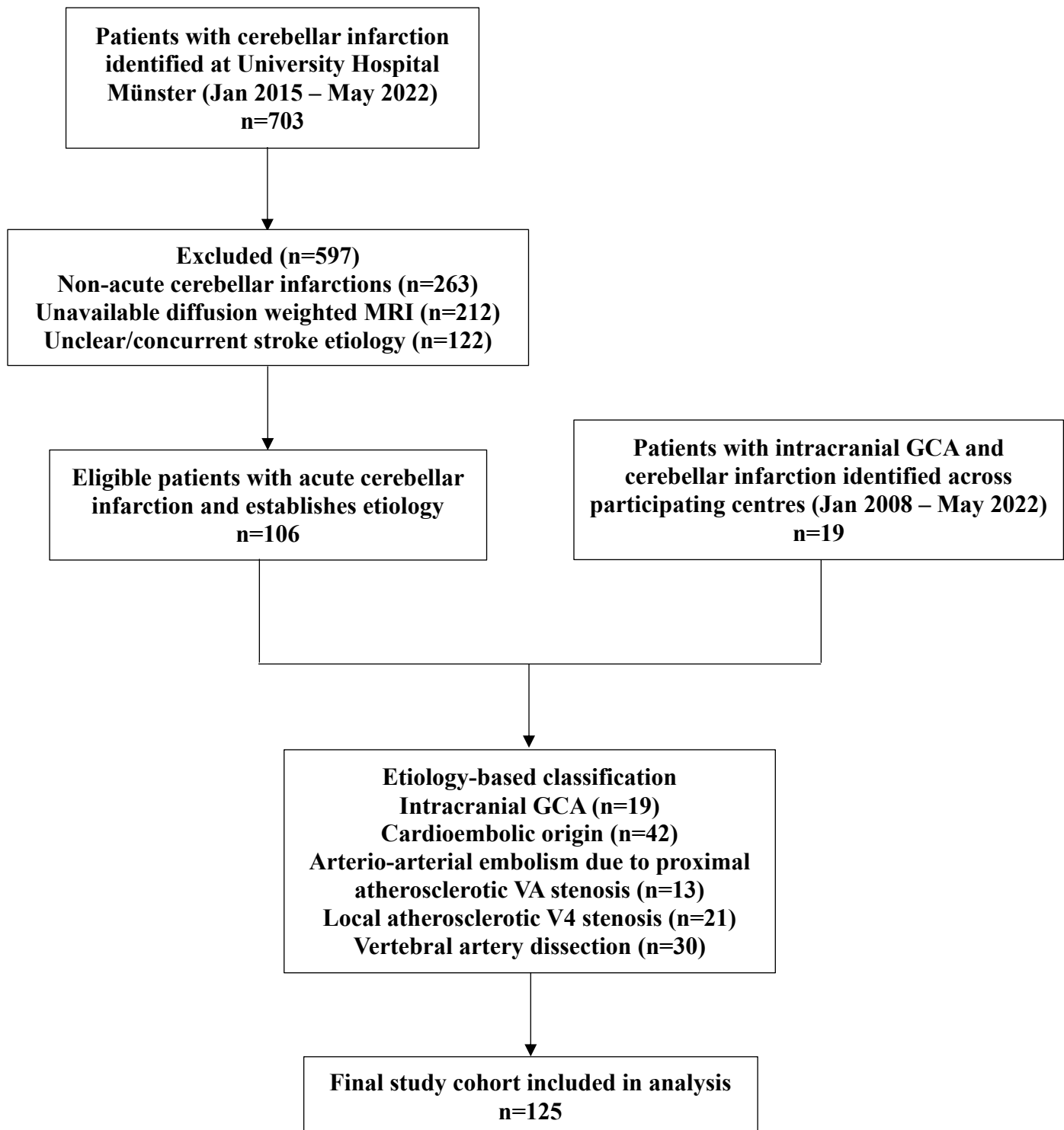

Supplement: sj-pdf-1-tan-10.1177_17562864251405203 – Supplemental material for A corona-like distribution and patchy pattern of cerebellar infarcts identify patients with giant cell arteritis [file sj-pdf-1-tan-10.1177_17562864251405203.pdf]
